# Application of Long-Read Whole-Genome Sequencing to Clarify Genotypic–Phenotypic Discrepancies in Methicillin-Resistant *Staphylococcus aureus*

**DOI:** 10.3390/diagnostics16081240

**Published:** 2026-04-21

**Authors:** Jin Ho Jhang, Kwangjin Ahn, Dokyun Kim, Seok Hoon Jeong, Hyun Soo Kim, Young Ree Kim, Young Ah Kim, Kyeong Seob Shin, Jeong Hwan Shin, Jeong Su Park, Kyoung Un Park, Yong Jun Kwon, Soo Hyun Kim, Jong Hee Shin, Soon Young Ahn, Sung Young Lee, Song-mee Bae, Jung Sik Yoo, Young Uh

**Affiliations:** 1 Department of Laboratory Medicine, Yonsei University Wonju College of Medicine, 20, Ilsan-ro, Wonju-si 26426, Republic of Korea; jjhskywalker@gmail.com (J.H.J.); kjahn123@yonsei.ac.kr (K.A.); 2 Department of Laboratory Medicine, Research Institute of Bacterial Resistance, Yonsei University College of Medicine, 211 Eonju-ro, Gangnam-gu, Seoul 06273, Republic of Korea; kyunsky@yuhs.ac (D.K.); kscpjsh@yuhs.ac (S.H.J.); 3 Department of Laboratory Medicine, Hallym University Dongtan Sacred Heart Hospital, Hallym University College of Medicine, 7, Keunjaebong-gil, Hwaseong-si 18450, Republic of Korea; hskim0901@empas.com; 4 Department of Laboratory Medicine, Jeju National University College of Medicine, 102, Jejudaehak-ro, Jeju-si 63243, Republic of Korea; namu8790@jejunu.ac.kr; 5 Department of Laboratory Medicine, National Health Insurance Service, Ilsan Hospital, 100, Ilsan-ro, Ilsandong-gu, Goyang-si 10444, Republic of Korea; yakim@nhimc.or.kr; 6 Department of Laboratory Medicine, Chungbuk National University College of Medicine, 776, 1sunhwan-ro, Seowon-gu, Cheongju-si 28644, Republic of Korea; ksshin@chungbuk.ac.kr; 7 Department of Laboratory Medicine, Paik Institute for Clinical Research, Inje University College of Medicine, 75 75, Bokji-ro, Busanjin-gu, Busan 47392, Republic of Korea; jhsmile@paik.ac.kr; 8 Department of Laboratory Medicine, Seoul National University College of Medicine, Seoul National University Bundang Hospital, 82, Gumi-ro 173beon-gil, Bundang-gu, Seongnam-si 13620, Republic of Korea; mdmicrobe@gmail.com (J.S.P.);; 9 Department of Laboratory Medicine, Chonnam National University Medical School, 42, Jebong-ro, Dong-gu, Gwangju 61469, Republic of Koreaalpinboy@hanmail.net (S.H.K.); shinjh@jnu.ac.kr (J.H.S.); 10 Department of Laboratory Medicine, Wonju Severance Christian Hospital, 20, Ilsan-ro, Wonju-si 26426, Republic of Korea; violet00366@daum.net; 11 Division of Antimicrobial Resistance Research, National Institute of Health, Korea Disease Control and Prevention Agency, Osong Health Technology Administration Complex, 187, Osongsaengmyeong 2-ro, Osong-eup, Heungdeok-gu, Cheongju-si 28159, Republic of Korea; blueskyi7@korea.kr (S.Y.L.); songmee@korea.kr (S.-m.B.); 12 Division of Bacterial Disease Research, National Institute of Health, Korea Disease Control and Prevention Agency, Osong Health Technology Administration Complex, 187, Osongsaengmyeong 2-ro, Osong-eup, Heungdeok-gu, Cheongju-si 28159, Republic of Korea; jungsiku@naver.com

**Keywords:** *Staphylococcus aureus*, Kor-GLASS, methicillin-resistant *S. aureus*, staphylococcal cassette chromosome *mec*, whole-genome sequencing

## Abstract

**Background/Objectives**: The Korean Global Antimicrobial Resistance Surveillance System monitors bloodstream *Staphylococcus aureus* infections by combining antimicrobial susceptibility testing (AST) with conventional polymerase chain reaction (PCR). Considering the clinical significance of methicillin-resistant *S. aureus* (MRSA), we performed an in-depth analysis of isolates showing genotypic–phenotypic discrepancies. **Methods**: Isolates were collected from designated collection centers in the Republic of Korea between 2017 and 2024. The 30 μg cefoxitin disk diffusion method was used to define the phenotypes. PCR targeting *mecA* and the staphylococcal cassette chromosome *mec* (SCC*mec*) was used to identify genotypes through gel electrophoresis. Long-read whole-genome sequencing (WGS) was performed using the Revio system (Pacific Biosciences) for isolates exhibiting discrepancies between phenotypes and genotypes. **Results**: In total, 5808 isolates were screened, and seven cases of genotypic–phenotypic discrepancies were identified, including one infant and six elderly patients with chromosomal SCC*mec* type IV. Although WGS confirmed intact PCR primer-binding sites, structural alterations were observed: three isolates had normal-length *mecA* and *mecR1*, two had partial deletions in *mecA*, and two featured either *mecA* or *mecR1* split into two proteins. Notably, although the six isolates with intact *mecR1* genes matched the nucleotide length of SCC*mec* type IV, their sequences exhibited high homology with SCC*mec* type II. **Conclusions**: Despite the presence of *mecA*, the non-standard configuration of regulatory genes within the SCC*mec* elements suppressed actual resistance expression. Because conventional PCR focusing on partial gene segments could overlook such phenotypic traits, the meticulous observation and implementation of WGS are crucial for the accurate characterization of genotypic–phenotypic discrepancies.

## 1. Introduction

Since the introduction of penicillin, the emergence of methicillin-resistant *Staphylococcus aureus* (MRSA) has been a persistent public health challenge, imposing a significant clinical burden in both healthcare and community settings, highlighting the need to understand its epidemiology [1,2]. In clinical laboratories, MRSA detection commonly relies on cefoxitin susceptibility testing because cefoxitin strongly induces the expression of the *mecA* gene, ensuring accurate and reproducible detection [3]. Polymerase chain reaction (PCR)-based assays target *mecA* and offer higher sensitivity and shorter turnaround times than culture-based approaches [2,4]. These tests support the earlier initiation of appropriate therapy and infection control measures [2].

The accurate interpretation of methicillin resistance requires the consideration of *mecA* and the staphylococcal cassette chromosome *mec* (SCC*mec*), which surrounds the genomic context and regulatory elements [4,5]. The International Working Group on the Classification of Staphylococcal Cassette Chromosome Elements (IWG-SCC) has proposed standardized criteria for SCC*mec* classification [5]. Whole-genome sequencing (WGS) provides a comprehensive framework for characterizing MRSA by resolving the full SCC*mec* architecture [4]. Moreover, multiple genes, including *mecI*, *mecR1*, and *mecR2*, and other insertion elements have been implicated in *mecA* transcription [6]. However, PCR testing requires costly equipment, reagents, and specialized consumables. For laboratories processing high volumes of samples, culture-based methods remain an economically viable option, providing high accuracy at a fraction of the cost [7].

The Korea Global Antimicrobial Resistance Surveillance System (Kor-GLASS) conducts comprehensive surveillance of bloodstream *S. aureus* infections across 10 representative tertiary care hospitals in the Republic of Korea [8]. Given that approximately 700 isolates are processed annually, the efficient allocation of laboratory resources is of paramount importance [8]. Antimicrobial susceptibility testing (AST) has followed the Clinical and Laboratory Standards Institute (CLSI) guidelines, and genetic analysis of virulence factors and resistance genes has been performed using conventional electrophoresis-based PCR [3,8]. Because conventional PCR cannot capture the complete genomic structure, discrepancies may arise between phenotypic AST results and molecular genotypic findings [9]. While there have been sporadic reports of clinical isolates harboring the *mecA* gene yet displaying phenotypic susceptibility to cefoxitin or oxacillin [9,10,11,12,13], such atypical strains have been continuously identified within Kor-GLASS since its inception in 2016 [8]. In this study, we used WGS to investigate the genetic mechanisms underlying discordant methicillin-resistant phenotypes among *mecA*-positive blood isolates. Our findings aimed to improve the interpretation of routine molecular testing and provide genomic insights relevant to MRSA surveillance and clinical decision-making.

## 2. Materials and Methods

### 2.1. Bacterial Collection

This study analyzed bacterial isolates obtained through the Kor-GLASS between 2017 and 2024 [8]. To ensure national representativeness, the Korea Disease Control and Prevention Agency (KDCA) (Osong Health Technology Administration Complex, 187, Osongsaengmyeong 2-ro, Osong-eup, Heungdeok-gu, Cheongju-si 28159, Republic of Korea) implemented the phased expansion of the surveillance network, strategically increasing the number of participating sentinel centers over successive periods [8,14]. At the designated analysis center for *S. aureus*, we meticulously evaluated all isolates identified from blood cultures and transferred them to sentinel centers [3]. Upon arrival, we performed a secondary verification of each isolate using matrix-assisted laser desorption ionization time-of-flight mass spectrometry (MALDI-TOF MS) (Bruker Biotyper, Bruker Daltonics GmbH & Co., Bremen, Germany) to confirm taxonomic integrity [15].

To maintain the highest standards of patient confidentiality during this logistical transition, we enforced a strict anonymization protocol. While personally identifiable medical information remained strictly anonymized, supplemental microbiological data were obtained alongside isolate transfers to facilitate a comprehensive analysis [14]. We categorized the origin of each infection based on the timing of specimen collection relative to hospital admission; isolates identified from specimens collected within 48 h of admission were classified as being of community origin, whereas those recovered after the 48 h threshold were designated as being of hospital origin [8]. With the microbial isolates, we recorded the sex and birth year of each patient to determine their age at the time of identification. Age groups were defined as follows: infants (<1 year), children (1–12 years), adolescents (13–18 years), adults (19–64 years), and older adults (≥65 years).

### 2.2. Antimicrobial Susceptibility Testing

Antimicrobial susceptibility profiles were determined using the disk diffusion method, strictly adhering to standardized protocols and interpretative criteria established by the CLSI [3]. To ascertain methicillin resistance in *S. aureus*, a 30 μg cefoxitin disk was employed as a surrogate marker for the presence of the *mecA* gene [3]. The bacterial suspension was prepared using the direct colony suspension method. Colonies grown on Mueller–Hinton agar (BANDIO BioScience Co., Pocheon, Republic of Korea) for 18–24 h were suspended in sterile saline and adjusted to a 0.5 McFarland turbidity standard to achieve a confluent lawn of growth. Subsequent to the application of the cefoxitin disks, the plates were subjected to incubation at a controlled temperature of 33–35 °C for 16–18 h. The resulting zones of inhibition were measured meticulously to the nearest millimeter. According to the CLSI M100 criteria, isolates exhibiting an inhibition zone diameter of ≥22 mm were categorized as susceptible, whereas those with a diameter of ≤21 mm were designated as resistant [3].

### 2.3. Genotypic Screening Using Conventional PCR

Total genomic DNA was extracted from isolates using a modified enzymatic lysis protocol. Briefly, bacterial cells were harvested from 1000 μL of Luria–Bertani broth by centrifugation. The resulting pellet was resuspended in 180 μL of lysozyme (30 mg/mL), followed by incubation at 37 °C for 30 min. Subsequently, the cells were treated with 20 μL of proteinase K and 200 μL of lysis buffer, with sequential incubations at 56 °C for 30 min and 70 °C for 40 min. The purified DNA was then obtained through a spin-column procedure, involving absolute ethanol precipitation and a series of wash steps, before a final elution in 200 μL of AE buffer. The bacterial genomic DNA was extracted using the Exgene™ Cell SV mini kit (GeneAll Biotechnology Co., Seoul, Republic of Korea). The procedure strictly adhered to the manufacturer’s instructions for all components included in the kit, ensuring standardized lysis and extraction conditions.

Following extraction, *mecA* gene detection and SCC*mec* genotyping were performed via conventional PCR to amplify target sequences; the specific primer combinations and thermal cycling profiles are summarized in Table S1. We designed the *mecA*-specific primers to amplify a 300 bp nucleic acid fragment. Our design incorporated specific primers that differentiate the *mec* complex classes based on *mecA* and *mecR1* sequences. Simultaneously, the assay utilizes primers targeting the *ccr* complex, which bind selectively according to *ccrA* and *ccrB* classes (Table S1). This screening was conducted to verify the presence of resistance-associated genetic determinants prior to genomic structural analysis. The resulting amplicons were resolved by gel electrophoresis using a 1.5% agarose gel prepared by dissolving SeaKem LE Agarose (Lonza, Basel, Switzerland) in 0.5X TBE buffer (Bioneer, Daejeon, Republic of Korea) via microwave heating. The gel was stained with 5 μL of a Dyne Gel Safe Red Kit (Dyne Bio, Seongnam, Republic of Korea) and solidified in a casting tray. Electrophoresis was conducted using the Mupid-One system (Advance, Tokyo, Japan), and a 100 bp Plus DNA Ladder (Genepia, Seoul, Republic of Korea) was used as a molecular weight marker. DNA bands were visualized and captured using a Gel Doc XR System (Bio-Rad Laboratories, Hercules, CA, USA). We determined the SCC*mec* type by estimating the DNA band sizes using a molecular marker and confirming the specific types of *mec* and *ccr* gene complexes (Table S2).

### 2.4. PacBio Sequencing and Bioinformatic Pipeline

The isolates were sent to Macrogen (Seoul, Republic of Korea) for comprehensive genomic analysis. For sample preparation, the target strains were pure-cultured on three separate Blood Agar Plates (BANDIO BioScience Co.) to ensure a sufficient biomass for extracting the high-quality genomic nucleic acid required for long-read sequencing, and they were then sequenced. Whole-genome sequencing was performed using a Revio system (Pacific Biosciences, Menlo Park, CA, USA), a single-molecule real-time (SMRT) sequencing platform, to generate high-fidelity long reads. De novo assembly was performed using the Microbial Genome Analysis application (SMRTLINK v25.1; Pacific Biosciences) to construct circularized contiguous sequences. To ensure genomic integrity, the assembly was refined using Inspector v1.0.1 (Johns Hopkins University, Baltimore, MD, USA) to detect and correct structural errors. Gene prediction was conducted using Prokka v1.14.6 (Victorian Bioinformatics Consortium, Melbourne, Australia) to identify protein-coding sequences and RNA genes. Functional annotation was performed by aligning the predicted proteins against the Evolutionary Genome of Genes: Non-supervised Orthologous Groups (European Molecular Biology Laboratory, Heidelberg, Germany) and InterProScan (European Bioinformatics Institute, Hinxton, UK) databases to assign biological roles. The assembly was validated through Average Nucleotide Identity analysis using Pyani (University of Strathclyde, Glasgow, UK) and completeness assessment via Benchmarking Universal Single-Copy Orthologs (University of Geneva, Geneva, Switzerland). The final functional annotation results were confirmed using a comprehensive spreadsheet. This enabled precise identification of each gene’s specific contig location, genomic coordinates, and strand orientation. Furthermore, the retrieved sequences of the *mecA* gene and relevant cassette components were cross-verified using the public SCCmecFinder database https://cge.food.dtu.dk/services/SCCmecFinder/ (accessed on 12 January 2026).

## 3. Results

### 3.1. General Epidemiology of MRSA

Since 2017, Kor-GLASS has rigorously monitored *S. aureus* isolates, defining MRSA as those displaying phenotypic cefoxitin resistance, along with the molecular confirmation of the *mec* gene. A longitudinal analysis revealed that the overall prevalence of MRSA steadily declined from 53.2% in 2017 to 41.4% by 2024. Regarding the structural architecture, SCC*mec* types II and IV consistently dominated the population, with only three exceptions: two type III strains in 2018 and a single type VI strain in 2019. Furthermore, we classified the SCC*mec* type as “not determined” when the *mecA* or *mecC* PCR results did not align with the *mec* complex results or when we observed DNA band sizes that did not meet the established criteria (Table S2); a total of 74 MRSA fell into this category. Molecular epidemiology has shifted markedly over time, with type IV beginning to exert growing dominance in 2019. The proportion of MRSA among community-origin isolates never surpassed the 40% threshold, while those from hospital-origin isolates sustained an average of 61.2%. Men accounted for a higher proportion of MRSA cases in 2019, whereas women took the lead between 2020 and 2023, before men reclaimed the majority in 2024. Across diverse age groups, the burden manifested in a distinct hierarchy: infants exhibited the highest vulnerability at 67.0%, older adults followed at 50.7%, adults represented 39.0%, children represented 34.0%, and adolescents demonstrated the lowest incidence at 20.0% (Table 1).

### 3.2. Atypical Cefoxitin-Susceptible mecA-Positive Isolates

**Figure 1:**
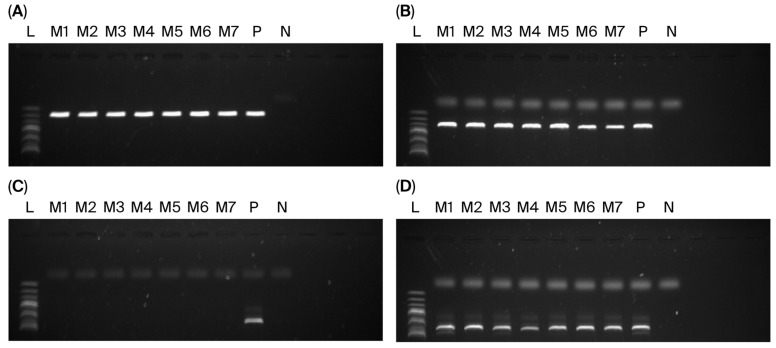
PCR gel electrophoresis results for SCC*mec* analysis. The *mecA* gene (**A**), *ccr* complex (**B**), Class A *mec* complex (**C**), and other *mec* complexes (**D**) appear in their respective panels. PCR for the Class A *mec* complex (**C**) employed *mecA* (univ.) and *mecR1* (**A**) primers described in Table S1, whereas the other *mec* complex (**D**) incorporated a mixture of the six remaining primers excluding *mecR1* (**A**). A 100-bp DNA ladder (L) occupies the leftmost lane, featuring intensified bands at 500 bp increments for reference. Lanes M1 through M7 follow in sequential order. Positive (P) and negative (N) controls were used to validate the amplification process.

Despite the overall decline in resistance, we identified seven isolates that met the standard definitions of MRSA. Phenotypically, every isolate exhibited clear susceptibility to cefoxitin according to CLSI guidelines, with inhibition zones measuring ≥22 mm for the 30 μg disk. However, molecular analysis revealed a striking contradiction: conventional PCR and subsequent electrophoretic band visualization consistently confirmed the presence of the *mecA* gene in all seven isolates. Further genotypic characterization identified the *ccr* gene complex as A2B2 and the *mec* gene complex as class B, leading to the definitive classification of SCC*mec* type IV (Figure 1, Table 2).

These unique strains, which appeared sporadically between 2018 and 2024, accounted for 0.3% (2018), 0.6% (2020), 0.3% (2023), and 0.8% (2024) of the total MRSA population. In terms of clinical background, all isolates from 2018 and 2024, along with one from 2020, were of community origin, whereas the remaining 2020 isolates and 2023 strains were of hospital origin. Notably, although the 2020 hospital-origin strain was recovered from an infant, all other isolates were identified in senior patients (Table 2).

### 3.3. Structural Defects of mecA Identified via Long-Read Sequencing

SCCmecFinder initially classified all strains as SCC*mec* type IV (Figures S1–S7). To validate the SCCmecFinder results, we compared the functional annotations of our long-read WGS data with the reference sequence of the first reported SCC*mec* type IV [16]. SCC*mec* was integrated into the chromosome in all seven isolates (Table 3). Key components, including IS*431*, *ccrB2*, and *ccrA2*, maintained lengths and sequences identical to those of the reference (Table 3). Notably, while the reference contains IS*1272* in the *mec* complex, our isolates featured a gene explicitly annotated as an “IS*1182* family member” at that locus. Although IS*1272* was initially characterized as a unique element of *S. haemolyticus*, subsequent phylogenetic analyses and standardized classification systems have formally assigned it to the IS*1182* family because of its high sequence homology with the family prototype [17]. SCCmecFinder correctly interpreted the IS*1182* family-associated sequence as an IS*1272* element. Regarding *mec* determinants, isolate M2 lacked mecA entirely, whereas isolates M1, M6, and M7 harbored truncated versions. Furthermore, M6 possessed a fragmented mecR1 that was split into two distinct segments (Table 3).

**Figure 2:**
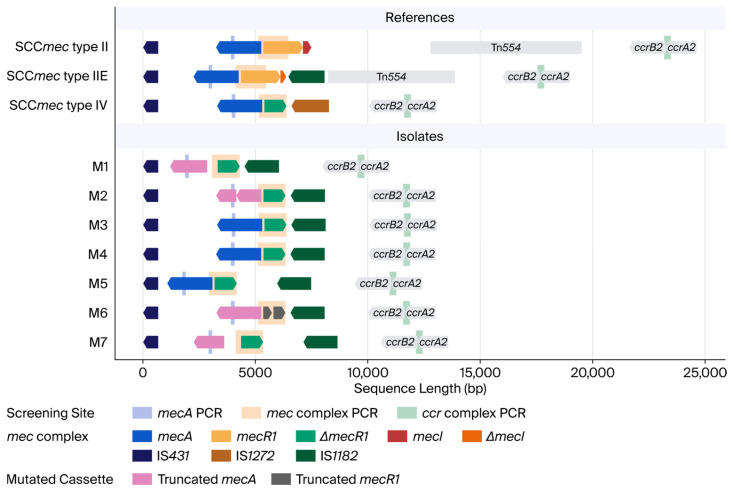
Structural analysis of isolates against the reference isolates. Arrows indicate the components of SCC*mec*, and squares in the background mark PCR segments for screening with the primers described in Table S1. The *x*-axis denotes the relative length starting from the first nucleotide of IS*431*, where the *mec* complex originated. SCC*mec* types II, IIE, and IV utilize the reference sequences D86934.2 [5,18], AJ810120 [18], and AB063172.2 [5], respectively. Gene names identify the specific cassettes outside a *mec* complex. Genes with mutations in the isolates are shown with distinct colors to indicate truncation.

Isolates M1 and M7 exhibited deletions at the 5′ terminus of *mecA*, while M6 harbored a shorter deletion at the 3′ end (Figure 2). Notably, M2 contained a mid-sequence deletion that fractured the protein, resulting in two separate fragments (Figure 2). Despite these truncations, all seven isolates retained the specific primer-binding regions used in the conventional PCR (Figure 2). The structural integrity of *mecR1* typically varies by SCC*mec* type; while SCC*mec* type II generally harbors a complete *mecR1*, SCC*mec* type IV is characterized by a truncated version, officially designated as *ΔmecR1* [5]. Our analysis revealed that the six isolates with non-fragmented *ΔmecR1* and the reference SCC*mec* type IV maintained an identical length of 987 bp with complete sequence uniformity (Table 3). All strains possessed the intact sequence of the *ΔmecR1* terminus. Although isolates M1 and M7 carried a truncated *mecA* gene, every PCR segment for screening the *mec* complex appeared clearly. The annotation of all isolates revealed the absence of the *mecI* gene but the presence of IS*1182* (Figure 2). This insertion sequence generally occurs in subtypes of the class A *mec* complex, specifically within SCC*mec* type II [18]. In contrast, SCC*mec* type IV typically carries IS*1272* [5,18]. Consequently, these seven strains possessed a *ΔmecR1* gene identical to SCC*mec* type IV, while the downstream genetic structure mirrored the subtypes found in SCC*mec* type II. Additionally, the isolates lacked Tn*554*, yet they retained the *ccrA2* and *ccrB2* genes shared by both SCC*mec* types II and IV (Figure 2).

## 4. Discussion

Oxacillin-susceptible *mecA*-positive *S. aureus* (OS-MRSA) is prevalent worldwide. In population-based studies, OS-MRSA has been reported at low but measurable frequencies, including 1.8% in China and 3.25% among bloodstream isolates in Brazil [10,11]. Additional case reports from Spain and Indonesia further support its international distribution [12,13]. In our cohort, the lower prevalence (0.3%) observed may reflect the fact that our analysis was restricted to bloodstream infections rather than including non-blood clinical specimens, where OS-MRSA may be detected more frequently. A study from Indonesia suggested that the prevalence of OS-MRSA may vary by specimen type, with a higher proportion detected in non-blood specimens [13]. Therefore, OS-MRSA may also be more frequently identified in non-blood clinical specimens in Korea.

The expression of β-lactam resistance in MRSA is a complex regulatory process that cannot be fully explained solely by the presence of the *mecA* gene [1,2,4]. In the canonical SCC*mec* structure, the expression of *mecA* is controlled by the signaling protein MecR1 and the repressor MecI [2,6]. While SCC*mec* type II typically harbors both *mecR1* and *mecI*, type IV is characterized by the absence of *mecI* and a truncated *mecR1* frequently associated with the insertion sequence IS*1272* [5,16,17]. The seven isolates in this study carry a *ΔmecR1* identical to SCC*mec* type IV, rather than the typical type II. These strains also harbor IS*1182*, a genetic element characteristic of SCC*mec* type IIE [18], instead of the IS*1272* usually found in type IV. Although Shore et al. suggest that IS*1182* lacks a specific protein-coding function within the SCC*mec*, this enhanced resistance is achieved by disrupting or inserting into *mecI*. Interestingly, despite possessing intact *mecA* and *ΔmecR1* genes, isolates M3, M4, and M5 failed to exhibit phenotypic resistance. We hypothesize that the structural recombination between SCC*mec* type II and type IV elements suppresses *mecA* expression in these specific isolates. This discrepancy leads to the misclassification of these strains as methicillin-susceptible in routine clinical screenings that omit genetic testing. Consequently, these “stealth MRSA” strains pose a severe risk to public health by facilitating the undetected spread of resistance genes, necessitating urgent molecular follow-up investigations.

Classifying such isolates as methicillin-susceptible *S. aureus* based solely on phenotypic tests poses a significant clinical risk, as “stealth” *mecA* carriers can potentially overcome antibiotic pressure and trigger resistance breakthrough [9]. Despite the lack of phenotypic expression caused by regulatory dysfunction, the presence of a functional *mecA* genotype allows for the horizontal dissemination of antimicrobial resistance to heterologous species [19]. This discovery underscores the ongoing evolutionary adaptation of bacteria to human antimicrobial interventions and presents a critical challenge for future resistance management strategies [1,17].

The primary limitation of this study was the lack of epidemiological traceability of the patients identified. Although the discrepant isolates were recovered from highly vulnerable groups (one infant and six older adults), the cross-sectional nature of our isolate collection precluded a detailed analysis of their clinical origins. Consequently, determining whether these strains originated in hospital or community settings remains challenging, limiting our assessment of their broader epidemiological impact and transmission dynamics. Another limitation was that the hospitals selected as collection sites in the Republic of Korea were tertiary hospitals, which may not fully represent community-level transmission. Nevertheless, the fact that such trends were identified within this sample group alone raises awareness regarding the potential spread in the community and highlights the necessity of further investigation.

## 5. Conclusions

In this study, we successfully identified three definitive “stealth MRSAs”—isolates carrying the *mecA* gene without phenotypic expression—out of seven suspected candidates. Detailed genomic analysis revealed a unique genetic recombination in which the mecR1 gene exhibited SCC*mec* type IV, yet concurrently harbored the IS*1182* element characteristic of SCC*mec* type IIE. Identifying such complex structural variations through conventional gel electrophoresis-based screening would require an impractical number of specific primers and extensive labor, whereas the WGS workflow provided rapid, comprehensive insights. While Kor-GLASS operates as a national sentinel system to predict and counteract AMR disasters, this system must yield representative data while balancing comprehensive surveillance against limited resource allocation. Our findings underscore that WGS is not merely a matter of cost-efficiency but an essential competency for national monitoring programs to detect hidden threats of stealth MRSA [20,21]. Although long-read WGS offers high-resolution insights into these atypical strains, its current cost remains 31.2 times higher than conventional methods, primarily due to extensive annotation processes. By streamlining the workflow to skip full annotation and focus on sequence analysis, laboratories can reduce these expenses by approximately one-third. Ultimately, establishing the analytical pipeline that accurately identifies these genetic-phenotypic discrepancies is the next step to integrating these high-resolution analytical capabilities into the modern era of rapid global AMR transmission.

## Figures and Tables

**Table 1:** Detailed prevalence of methicillin-resistant *Staphylococcus aureus* (MRSA) cases.

Details	No. of Isolates (Proportions, %)								
	2017	2018	2019	2020	2021	2022	2023	2024	Total
MRSA	377/708	349/742	331/677	348/733	349/774	335/734	374/827	254/613	2717/5808
	(53.2)	(47.0)	(48.9)	(47.5)	(45.1)	(45.6)	(45.2)	(41.4)	(46.8)
SCC*mec* type II ^1^	186 (49.3)	176 (50.4)	140 (42.3)	130 (37.4)	118 (33.8)	110 (32.8)	112 (29.9)	66 (26.0)	1038 (38.2)
SCC*mec* type IV ^1^	186 (49.3)	159 (45.6)	185 (55.9)	209 (60.1)	222 (63.6)	217 (64.8)	251 (67.1)	173 (68.1)	1602 (59.0)
MRSA by origin									
Community	139/348	135/417	143/385	154/424	150/424	135/421	196/509	123/360	1175/3288
	(39.9)	(32.4)	(37.1)	(36.3)	(35.4)	(32.1)	(38.5)	(34.2)	(35.7)
Hospital	238/360	214/325	188/292	194/309	199/350	200/313	178/318	131/253	1542/2520
	(66.1)	(65.8)	(64.4)	(62.8)	(56.9)	(63.9)	(56.0)	(51.8)	(61.2)
MRSA by sex									
Male	228/423	201/428	204/407	189/423	201/458	183/429	218/505	153/357	1577/3430
	(53.9)	(47.0)	(50.1)	(44.7)	(43.9)	(42.7)	(43.2)	(42.9)	(46.0)
Female	149/285	148/314	127/270	159/310	148/316	152/305	156/322	101/256	1140/2378
	(52.3)	(47.1)	(47.0)	(51.3)	(46.8)	(49.8)	(48.4)	(39.5)	(47.9)
MRSA by Age ^2^									
Infants	15/22	12/15	6/16	17/20	15/24	8/12	1/3	3/3	77/115
	(68.2)	(80.0)	(37.5)	(85.0)	(62.5)	(66.7)	(33.3)	(100)	(67.0)
Children	7/20	3/14	6/12	5/14	3/6	3/10	2/7	3/11	32/94
	(35.0)	(21.4)	(50.0)	(35.7)	(50.0)	(30.0)	(28.6)	(27.3)	(34.0)
Adolescents	3/10	1/10	0/2	2/5	0/6	1/5	2/8	1/4	10/50
	(30.0)	(10.0)	(0)	(40.0)	(0)	(20.0)	(25.0)	(25.0)	(20.0)
Adults	109/237	88/249	112/227	88/236	95/252	85/224	79/248	64/171	720/1844
	(46.0)	(35.3)	(49.3)	(37.3)	(37.7)	(37.9)	(31.9)	(37.4)	(39.0)
Seniors	243/419	245/454	207/420	236/458	236/486	238/483	290/561	183/424	1878/3705
	(58.0)	(54.0)	(49.3)	(51.5)	(48.6)	(49.3)	(51.7)	(43.2)	(50.7)

^1^ This value represents the proportions of SCC*mec* types II and IV among the total MRSA isolates. ^2^ Age categories were defined as follows: infants, <1 year; children, 1–12 years; adolescents, 13–18 years; adults, 19–64 years; and older adults, ≥65 years. Abbreviations: SCC, staphylococcal cassette chromosome.

**Table 2:** Seven cefoxitin-susceptible *mecA* carriers.

Identification			Patient Information		Zone Diameter of Cefoxitin Disk Diffusion Test (Interpretation)	Results of Conventional PCR		
Isolates	Year	Origin	Sex	Age (yr)		*mecA*	*mecC*	SCC*mec* Type
M1	2018	Community	M	81	30 mm (S)	P	N	IV
M2	2020	Community	M	65	30 mm (S)	P	N	IV
M3	2020	Hospital	F	0	25 mm (S)	P	N	IV
M4	2023	Hospital	F	70	25 mm (S)	P	N	IV
M5	2024	Community	F	75	25 mm (S)	P	N	IV
M6	2024	Community	F	70	30 mm (S)	P	N	IV
M7	2024	Community	F	80	27 mm (S)	P	N	IV

Abbreviations: S, susceptible; PCR, polymerase chain reaction; SCC, staphylococcal cassette chromosome.

**Table 3:** Comparison of SCC*mec* type IV components between isolates and reference.

Isolate	Location of SCC*mec*	Lengths of SCC*mec* Components (bp)					
		IS*431*	*mecA*	*mecR1*	IS*1272* ^2^	*ccrB2*	*ccrA2*
Reference ^1^	Chromosome	675	2007	987	1659	1629	1350
M1	Chromosome	675	1644	987	1524	1629	1350
M2 ^3^	Chromosome	675	-	987	1524	1629	1350
M3	Chromosome	675	2007	987	1524	1629	1350
M4	Chromosome	675	2007	987	1524	1629	1350
M5	Chromosome	675	2007	987	1512	1629	1350
M6 ^4^	Chromosome	675	1998	384, 525	1524	1629	1350
M7	Chromosome	675	1350	987	1512	1629	1350

^1^ Reference sequence of the SCC*mec* type IV element from the originally reported strain (GenBank: AB063172.2). ^2^ Although the reference isolate contained IS*1272*, functional annotation of the seven isolates identified them as members of the IS*1182* family, resulting in lengths that did not exactly match the reference. ^3^ Functional annotation of the SCC*mec* element revealed the complete absence of *mecA*. ^4^ *mecR1* genes within the SCC*mec* element appeared to be fragmented into two distinct segments in the functional annotation. Abbreviations: SCC, staphylococcal cassette chromosome; BP, base pair; IS, insertion sequence.

## Data Availability

As data were de-identified and gathered as part of the World Health Organization Global Antimicrobial Resistance and Use Surveillance System (GLASS) expansion project, a direct analysis of the primary records could not be conducted. Nevertheless, a comprehensive overview of the Kor-GLASS initiative was obtained from annual surveillance reports issued by the Korea Disease Control and Prevention Agency (KDCA).

## Supplementary Materials

The following supporting information can be downloaded at: https://www.mdpi.com/article/10.3390/diagnostics16081240/s1, Table S1: Oligonucleotide primers and thermal cycling conditions; Table S2: SCC*mec* typing criteria based on electrophoretic band analysis; Figure S1: SCCmecFinder report for M1; Figure S2: SCCmecFinder report for M2; Figure S3: SCCmecFinder report for M3; Figure S4: SCCmecFinder report for M4; Figure S5: SCCmecFinder report for M5; Figure S6: SCCmecFinder report for M6; Figure S7: SCCmecFinder report for M7.
