# Supplementary material for: Application of Long-Read Whole-Genome Sequencing to Clarify Genotypic–Phenotypic Discrepancies in Methicillin-Resistant Staphylococcus aureus
Source: Diagnostics (Basel). 2026 Apr 21;16(8):1240. doi: 10.3390/diagnostics16081240 (PMC13114432; doi:10.3390/diagnostics16081240)
Supplement: Supplementary file 1 [file diagnostics-16-01240-s001.zip › Supplement for Application of Long-Read Whole-Genome Sequencing to Clarify Genotypic–Phenotypic Discrepancies in Methicil-lin-Resistant Staphylococcus aureus.pdf]

Table S1. Oligonucleotide primers and thermal cycling conditions.

Target gene	Primer name	Sequence(5′→3′)	Thermal cycle
mecA	*mecA*-P4	TCCAGATTACAACTTCACCAGG	94°C 5min +
	mecA-P7	CCACTTCATATCTTGTAACG	94°C 30sec, 59°C 1min,
mecC	*mecC* MFP	GAAAAAAAGGCTTAGAACGCCTC	72°C 30sec (30 times) +
	mecC MRP	GAAGATCTTTTCCGTTTTCAGC	72°C 10min
mec gene complex	*mecA*(univ.)-149–126	CTGCTATCTTTATAAACTTGTTTG	94°C 5min +
	mecA(E)-758–737	ACATAACCTAAAAGGTGTACTG	94°C 10sec, 56°C 20sec,
	mecR1(B)-987–966	TCATGTGAAGCTCGATATACTA	72°C 1min (28 times) +
	mecR1(E)-121–98	ACCAAACTTTATGATTTAACTGAG	72°C 7min
	mecR1(A)-1110–1087	TTCATTATAAAGCACAAAACTTCC	
	IS431(C2)-103–82	GTTGAATGATGAACGTTTACAC	
	IS431(C1)-254–275	GGGACATACATTAGATATTTGG	
ccr gene complex	*ccrA1*-609–628	CAGCCTTATCAGGTACGAA	94°C 5min +
	ccrA2-1136–1155	CATTACGTCAACAACCGCAA	94°C 10sec, 56°C 20sec,
	ccrA3-1106–1127	CTGAATCATTCAGAAAACAGAC	72°C 40sec (28 times) +
	ccrA4-1121–1141	CTCCTAAAACAGCAACAAATGA	72°C 7min
	ccrB1-375–356	GAGCATTAACTTGCCTGTTG	
	ccrB2-72–51	CCTTCTGTGCTTTGCATTTC	
	ccrB3-223–204	GACCTTCGTTCGCATCTTTT	
	ccrB4-68–49	GGTTACAGTATTCAAGGTCAAT	
	ccrB6-505–481	CTGGTATGTTATTATATCCTAAAG	
	ccrC-181–202	GCAATGAAACGTCTATTACAAG	
	ccrC-382–361	CAAACATTGTAACGAGTACTTC	

Table S2. SCC*mec* typing criteria based on electrophoretic band analysis.

SCC*mec* type	Size (bp)		*ccr*/*mec* gene combination
	ccr gene complex	*mec* gene complex	
I	672	1,235	A1B1/class B
II	307	1,358	A2B2/class A
III	982	1,358	A3B3/class A
IV	307	1,235	A2B2/class B
V	202	533	C/class C2
VI	406	1,235	A4B4/class B
VII	202	717	C/class C1
VIII	406	1,358	A4B4/class A
IX	672	533	A1B1/class C2
X	519	717	A1B6/class C1
XI	802	979	A1B3/class E

Abbreviations: SCC, staphylococcal chromosomal cassette

Table S3. Detailed prevalence and distribution of methicillin-resistant *Staphylococcus aureus* (MRSA) isolates

Details	Precise proportions								
	2017	2018	2019	2020	2021	2022	2023	2024	Total
MRSA	377/708	349/742	331/677	348/733	349/774	335/734	374/827	254/613	2,717/5,808
(S2/S4)[1]	(186/186)	(176/159)	(140/185)	(130/209)	(118/222)	(110/217)	(112/251)	(66/173)	(1,038/1,602)
MRSA by origin									
Community	139/348	135/417	143/385	154/424	150/424	135/421	196/509	123/360	1,175/3.288
Hospital	238/360	214/325	188/292	194/309	199/350	200/313	178/318	131/253	1,542/2,520
MRSA by sex									
Male	228/423	201/428	204/407	189/423	201/458	183/429	218/505	153/357	1,577/3,430
Female	149/285	148/314	127/270	159/310	148/316	152/305	156/322	101/256	1,140/2,378
MRSA by Ages[2]									
Infants	15/22	12/15	6/16	17/20	15/24	8/12	1/3	3/3	77/115
Children	7/20	3/14	6/12	5/14	3/6	3/10	2/7	3/11	32/94
Adolescents	3/10	1/10	0/2	2/5	0/6	1/5	2/8	1/4	10/50
Adults	109/237	88/249	112/227	88/236	95/252	85/224	79/248	64/171	720/1,844
Seniors	243/419	245/454	207/420	236/458	236/486	238/483	290/561	183/424	1,878/3,705

[1] This values represent each number of isolates of SCC*mec* type II (S2) and IV (S4) among the total MRSA isolates. [2] Age categories are defined as infants, <1 year; children, 1–12 years; adolescents, 13–18 years; adults, 19–64 years; and seniors, ≥65 years. Abbreviations: SCC, staphylococcal cassette chromosome.

A

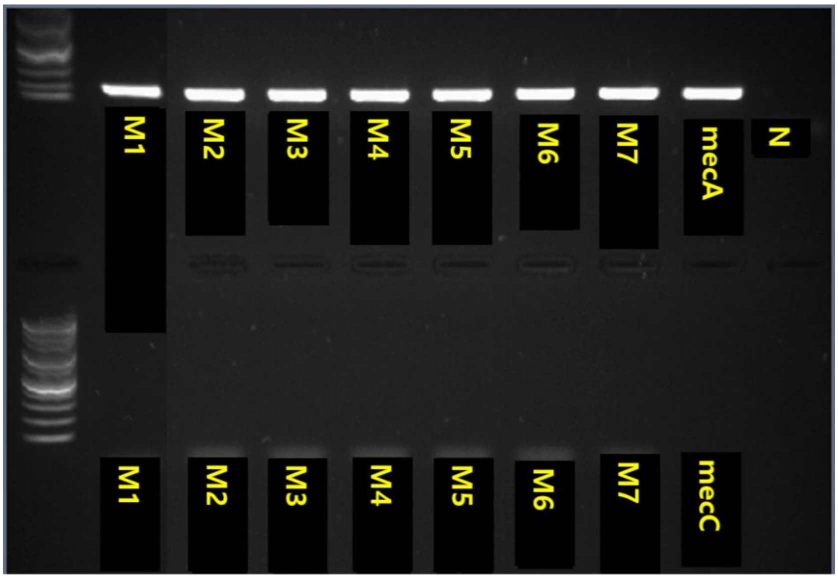

B

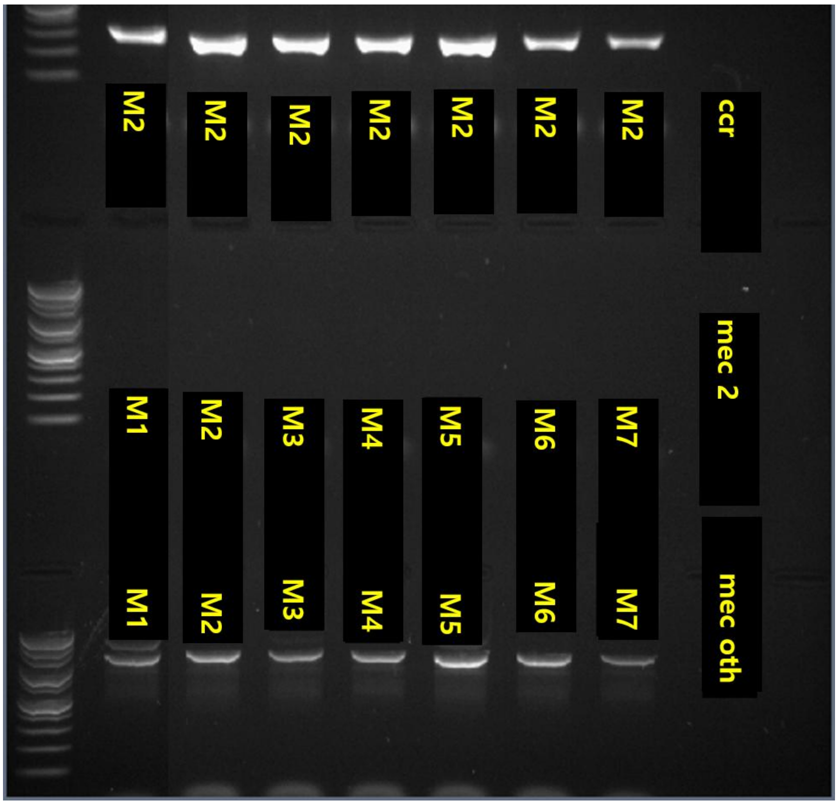

Figure S1. Electrophoretic analysis of *mec* and *ccr* gene complexes for SCC*mec* typing. A 100-bp DNA ladder is located on the left side of each gel, with intense reference bands positioned at 500-bp intervals to facilitate size estimation. (A) Lanes M1–M7 represent the seven studied isolates, all of which exhibit a clear band for *mecA* gene. Lane N serves as the negative control, while lanes labeled *mecA* and *mecC* indicate the respective positive controls. (B) The upper bands represent *ccr* gene complex. Due to the identical *ccr* band sizes (307 bp) and similar *mec* complex sizes (approx. 1,200–1,300 bp) between SCC*mec* types II and IV (Table S1), typing was performed in a sequential two-step process. Initial screening targeted SCC*mec* type II (mec 2), followed by a second PCR using a primer pool for other types (mec oth) to definitively classify the isolates.

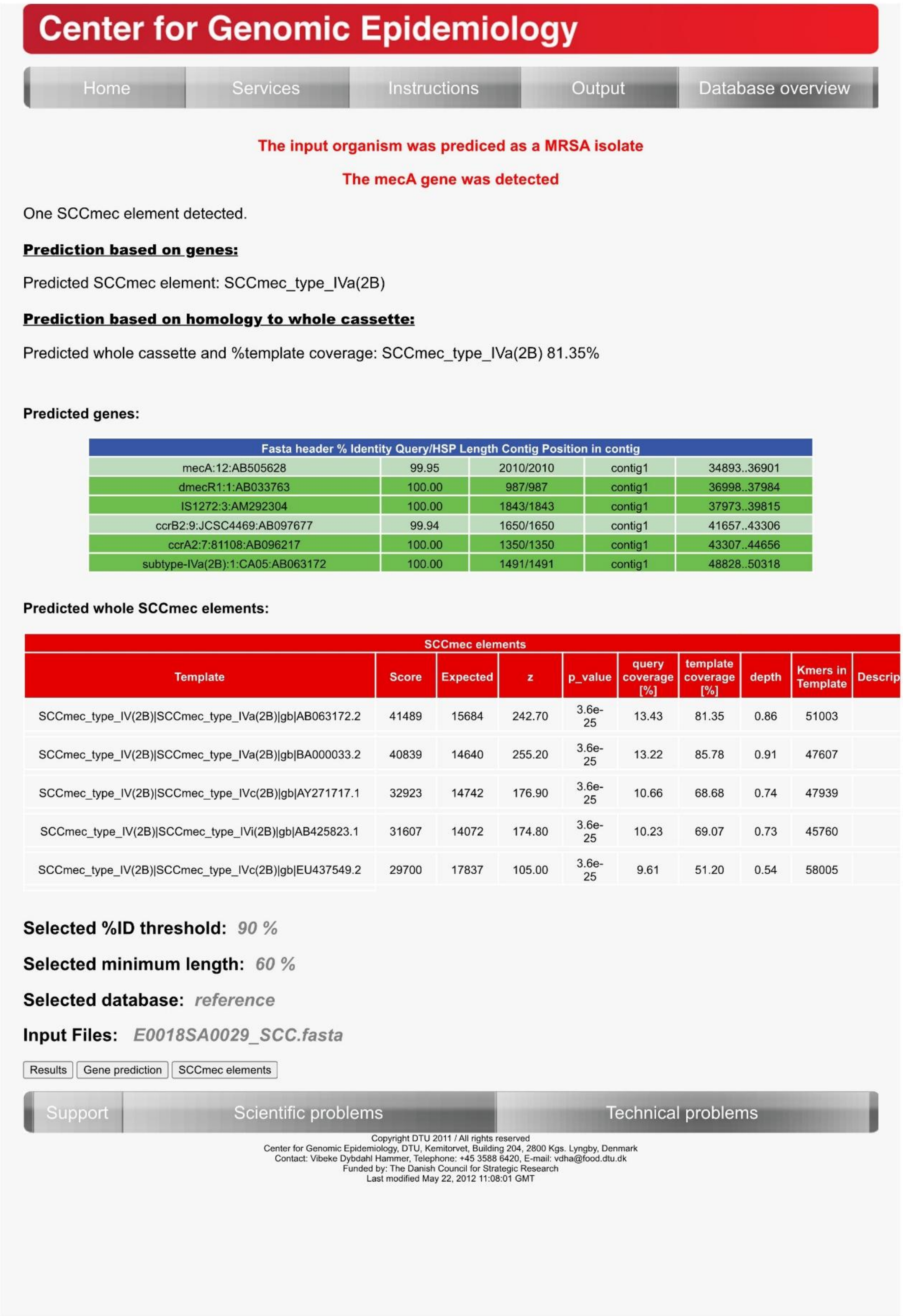

Center for Genomic Epidemiology

Home | Services | Instructions | Output | Database overview

The input organism was prediced as a MRSA isolate

The mecA gene was detected

One SCCmec element detected.

Prediction based on genes:

Predicted SCCmec element: SCCmec_type_IVa(2B)

Prediction based on homology to whole cassette:

Predicted whole cassette and %template coverage: SCCmec_type_IVa(2B) 81.35%

Predicted genes:

Fasta header	% Identity	Query/HSP Length	Contig	Position in contig
mecA:12:AB505628	99.95	2010/2010	contig1	34893..36901
dmecR1:1:AB033763	100.00	987/987	contig1	36998..37984
IS1272:3:AM292304	100.00	1843/1843	contig1	37973..39815
ccrB2:9:JCSC4469:AB097677	99.94	1650/1650	contig1	41657..43306
ccrA2:7:81108:AB096217	100.00	1350/1350	contig1	43307..44656
subtype-IVa(2B):1:CA05:AB063172	100.00	1491/1491	contig1	48828..50318

Predicted whole SCCmec elements:

SCCmec elements									
Template	Score	Expected	z	p_value	query coverage [%]	template coverage [%]	depth	Kmers in Template	Descrip
SCCmec_type_IV(2B)|SCCmec_type_IVa(2B)|gb|AB063172.2	41489	15684	242.70	3.6e-25	13.43	81.35	0.86	51003	
SCCmec_type_IV(2B)|SCCmec_type_IVa(2B)|gb|BA000033.2	40839	14640	255.20	3.6e-25	13.22	85.78	0.91	47607	
SCCmec_type_IV(2B)|SCCmec_type_IVc(2B)|gb|AY271717.1	32923	14742	176.90	3.6e-25	10.66	68.68	0.74	47939	
SCCmec_type_IV(2B)|SCCmec_type_IVi(2B)|gb|AB425823.1	31607	14072	174.80	3.6e-25	10.23	69.07	0.73	45760	
SCCmec_type_IV(2B)|SCCmec_type_IVc(2B)|gb|EU437549.2	29700	17837	105.00	3.6e-25	9.61	51.20	0.54	58005	

Selected %ID threshold: *90 %*

Selected minimum length: *60 %*

Selected database: *reference*

Input Files: *E0018SA0029_SCC.fasta*

Results | Gene prediction | SCCmec elements

Support | Scientific problems | Technical problems

Copyright DTU 2011 / All rights reserved
Center for Genomic Epidemiology, DTU, Kemitorvet, Building 204, 2800 Kgs. Lyngby, Denmark
Contact: Vibeke Dybdahl Hammer, Telephone: +45 3588 6420, E-mail: vdha@food.dtu.dk
Funded by: The Danish Council for Strategic Research
Last modified May 22, 2012 11:08:01 GMT

Figure S2. SCCmecFinder report of M1.

Center for Genomic Epidemiology

Home | Services | Instructions | Output | Database overview

The input organism was prediced as a MRSA isolate

The mecA gene was detected

One SCCmec element detected.

Prediction based on genes:

Predicted SCCmec element: SCCmec_type_IVa(2B)

Prediction based on homology to whole cassette:

Predicted whole cassette and %template coverage: SCCmec_type_IVa(2B) 88.78%

Predicted genes:

Fasta header	% Identity	Query/HSP Length	Contig	Position in contig
mecA:12:AB505628	99.95	2011/2010	contig1	39163..41173
dmecR1:1:AB033763	100.00	987/987	contig1	41270..42256
IS1272:3:AM292304	100.00	1843/1843	contig1	42245..44087
ccrB2:9:JCSC4469:AB097677	99.94	1650/1650	contig1	45929..47578
ccrA2:7:81108:AB096217	100.00	1350/1350	contig1	47579..48928
subtype-IVa(2B):1:CA05:AB063172	100.00	1491/1491	contig1	53045..54535

Predicted whole SCCmec elements:

SCCmec elements										
Template	Score	Expected	z	p_value	query coverage [%]	template coverage [%]	depth	Kmers in Template	Descrip
SCCmec_type_IV(2B)|SCCmec_type_IVa(2B)|gb|AB063172.2	45279	18165	246.40	3.6e-25	11.39	88.78	0.93	51003	
SCCmec_type_IV(2B)|SCCmec_type_IVa(2B)|gb|BA000033.2	44597	16955	260.10	3.6e-25	11.22	93.68	0.99	47607	
SCCmec_type_IV(2B)|SCCmec_type_IVc(2B)|gb|AY271717.1	36557	17073	183.00	3.6e-25	9.20	76.26	0.82	47939	
SCCmec_type_IV(2B)|SCCmec_type_IVi(2B)|gb|AB425823.1	35401	16297	183.80	3.6e-25	8.91	77.36	0.82	45760	
SCCmec_type_IV(2B)|SCCmec_type_IVj(2B)|gb|AB425824.1	32690	16466	155.40	3.6e-25	8.22	70.71	0.75	46234	

Selected %ID threshold: *90 %*

Selected minimum length: *60 %*

Selected database: *reference*

Input Files: *E0020SA0060_SCC.fasta*

Results | Gene prediction | SCCmec elements

Support | Scientific problems | Technical problems

Copyright DTU 2011 / All rights reserved
Center for Genomic Epidemiology, DTU, Kemitorvet, Building 204, 2800 Kgs. Lyngby, Denmark
Contact: Vibeke Dybdahl Hammer, Telephone: +45 3588 6420, E-mail: vdha@food.dtu.dk
Funded by: The Danish Council for Strategic Research
Last modified May 22, 2012 11:08:01 GMT

Figure S3. SCCmecFinder report of M2.

Center for Genomic Epidemiology

Home | Services | Instructions | Output | Database overview

The input organism was prediced as a MRSA isolate

The mecA gene was detected

One SCCmec element detected.

Prediction based on genes:

Predicted SCCmec element: SCCmec_type_IVa(2B)

Prediction based on homology to whole cassette:

Predicted whole cassette and %template coverage: SCCmec_type_IVa(2B) 91.22%

Predicted genes:

Fasta header	% Identity	Query/HSP Length	Contig	Position in contig
mecA:12:AB505628	100.00	2010/2010	contig1	42924..44933
dmecR1:1:AB033763	100.00	987/987	contig1	45030..46016
IS1272:3:AM292304	100.00	1843/1843	contig1	46005..47847
ccrB2:9:JCSC4469:AB097677	99.94	1650/1650	contig1	49689..51338
ccrA2:7:81108:AB096217	100.00	1350/1350	contig1	51339..52688
subtype-IVa(2B):1:CA05:AB063172	100.00	1491/1491	contig1	56860..58350

Predicted whole SCCmec elements:

SCCmec elements									
Template	Score	Expected	z	p_value	query coverage [%]	template coverage [%]	depth	Kmers in Template	Descript
SCCmec_type_IV(2B)|SCCmec_type_IVa(2B)|gb|AB063172.2	46525	17387	267.10	3.6e-25	11.82	91.22	0.99	51003	
SCCmec_type_IV(2B)|SCCmec_type_IVa(2B)|gb|BA000033.2	46021	16229	282.80	3.6e-25	11.70	96.67	1.05	47607	
SCCmec_type_IV(2B)|SCCmec_type_IVi(2B)|gb|AB425823.1	34277	15600	181.40	3.6e-25	8.71	74.91	0.83	45760	
SCCmec_type_IV(2B)|SCCmec_type_IVc(2B)|gb|AY271717.1	33027	16342	158.40	3.6e-25	8.39	68.89	0.77	47939	
SCCmec_type_IV(2B)|SCCmec_type_IVj(2B)|gb|AB425824.1	32771	15761	164.40	3.6e-25	8.33	70.88	0.79	46234	

Selected %ID threshold: *90 %*

Selected minimum length: *60 %*

Selected database: *reference*

Input Files: *I0020SA0059_SCC.fasta*

Results | Gene prediction | SCCmec elements

Support | Scientific problems | Technical problems

Copyright DTU 2011 / All rights reserved
Center for Genomic Epidemiology, DTU, Kemitorvet, Building 204, 2800 Kgs. Lyngby, Denmark
Contact: Vibeke Dybdahl Hammer, Telephone: +45 3588 6420, E-mail: vdha@food.dtu.dk
Funded by: The Danish Council for Strategic Research
Last modified May 22, 2012 11:08:01 GMT

Figure S4. SCCmecFinder report of M3.

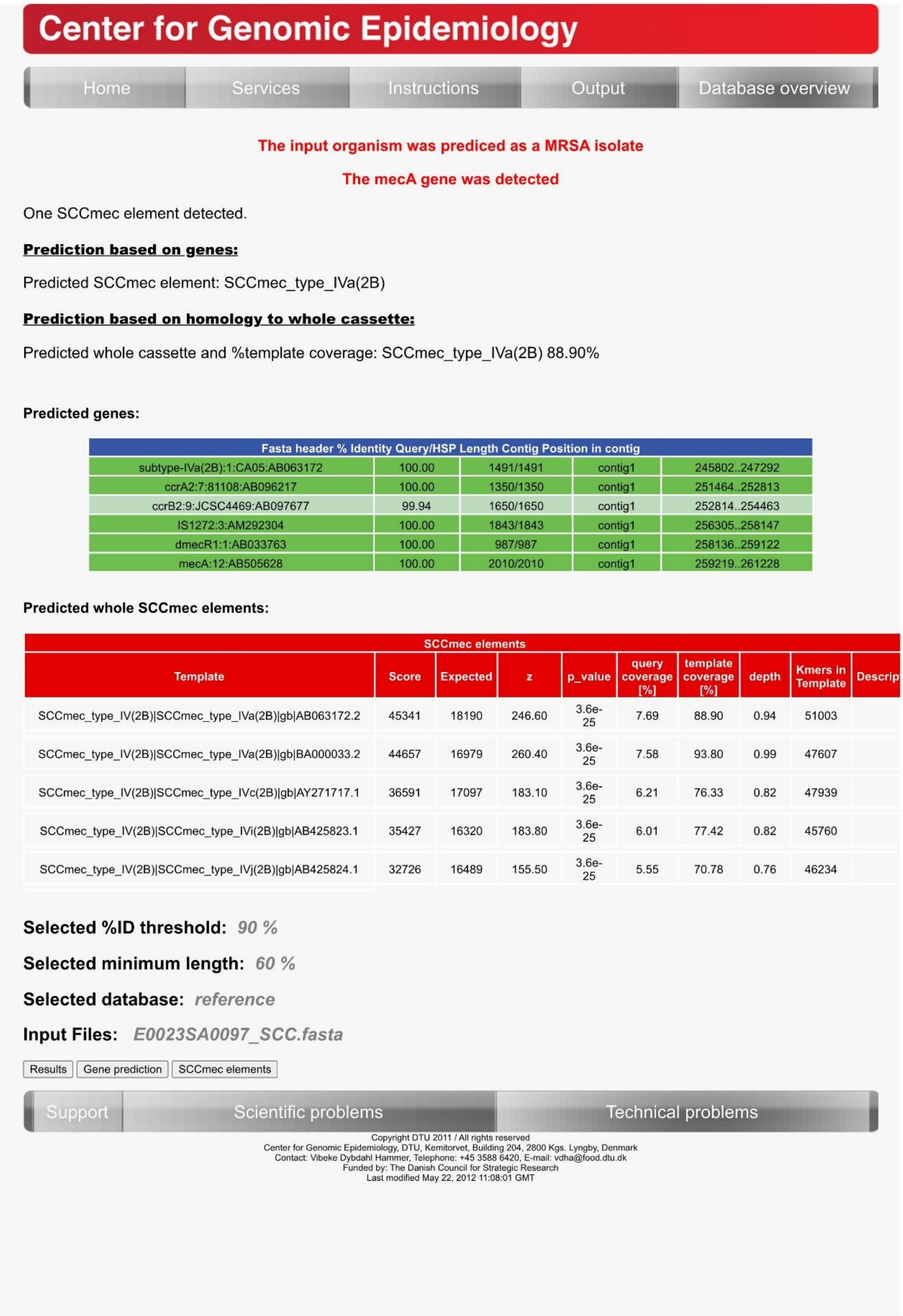

Center for Genomic Epidemiology

Home | Services | Instructions | Output | Database overview

The input organism was prediced as a MRSA isolate

The mecA gene was detected

One SCCmec element detected.

Prediction based on genes:

Predicted SCCmec element: SCCmec_type_IVa(2B)

Prediction based on homology to whole cassette:

Predicted whole cassette and %template coverage: SCCmec_type_IVa(2B) 88.90%

Predicted genes:

Fasta header	% Identity	Query/HSP Length	Contig	Position in contig
subtype-IVa(2B):1:CA05:AB063172	100.00	1491/1491	contig1	245802..247292
ccrA2:7:81108:AB096217	100.00	1350/1350	contig1	251464..252813
ccrB2:9:JCSC4469:AB097677	99.94	1650/1650	contig1	252814..254463
IS1272:3:AM292304	100.00	1843/1843	contig1	256305..258147
dmecR1:1:AB033763	100.00	987/987	contig1	258136..259122
mecA:12:AB505628	100.00	2010/2010	contig1	259219..261228

Predicted whole SCCmec elements:

SCCmec elements									
Template	Score	Expected	z	p_value	query coverage [%]	template coverage [%]	depth	Kmers in Template	Descrip
SCCmec_type_IV(2B)|SCCmec_type_IVa(2B)|gb|AB063172.2	45341	18190	246.60	3.6e-25	7.69	88.90	0.94	51003	
SCCmec_type_IV(2B)|SCCmec_type_IVa(2B)|gb|BA000033.2	44657	16979	260.40	3.6e-25	7.58	93.80	0.99	47607	
SCCmec_type_IV(2B)|SCCmec_type_IVc(2B)|gb|AY271717.1	36591	17097	183.10	3.6e-25	6.21	76.33	0.82	47939	
SCCmec_type_IV(2B)|SCCmec_type_IVi(2B)|gb|AB425823.1	35427	16320	183.80	3.6e-25	6.01	77.42	0.82	45760	
SCCmec_type_IV(2B)|SCCmec_type_IVj(2B)|gb|AB425824.1	32726	16489	155.50	3.6e-25	5.55	70.78	0.76	46234	

Selected %ID threshold: *90 %*

Selected minimum length: *60 %*

Selected database: *reference*

Input Files: *E0023SA0097_SCC.fasta*

Results | Gene prediction | SCCmec elements

Support | Scientific problems | Technical problems

Copyright DTU 2011 / All rights reserved
Center for Genomic Epidemiology, DTU, Kemitorvet, Building 204, 2800 Kgs. Lyngby, Denmark
Contact: Vibeke Dybdahl Hammer, Telephone: +45 3588 6420, E-mail: vdha@food.dtu.dk
Funded by: The Danish Council for Strategic Research
Last modified May 22, 2012 11:08:01 GMT

Figure S5. SCCmecFinder report of M4.

Center for Genomic Epidemiology

Home | Services | Instructions | Output | Database overview

The input organism was prediced as a MRSA isolate

The mecA gene was detected

One SCCmec element detected.

Prediction based on genes:

Predicted SCCmec element: SCCmec_type_IVc(2B)

Prediction based on homology to whole cassette:

Predicted whole cassette and %template coverage: SCCmec_type_IVc(2B) 83.94%

Predicted genes:

Fasta header	% Identity	Query/HSP Length	Contig	Position in contig
subtype-IVc(2B):3:81108:AB096217	100.00	1155/1155	contig1	134710..135864
ccrA2:4:cm11:EU437549	100.00	1350/1350	contig1	139881..141230
ccrB2:4:cm11:EU437549	100.00	1629/1629	contig1	141252..142880
IS1272:2:AB033763	100.00	1585/1585	contig1	144722..146306
dmecR1:1:AB033763	100.00	987/987	contig1	148113..149099
mecA:12:AB505628	100.00	2010/2010	contig1	149196..151205

Predicted whole SCCmec elements:

SCCmec elements									
Template	Score	Expected	z	p_value	query coverage [%]	template coverage [%]	depth	Kmers in Template	Descrip
SCCmec_type_IV(2B)|SCCmec_type_IVc(2B)|gb|EU437549.2	48690	17116	280.50	3.6e-25	13.15	83.94	0.82	58005	
SCCmec_type_IV(2B)|SCCmec_type_IVc(2B)|gb|AY271717.1	37383	14146	228.30	3.6e-25	10.10	77.98	0.78	47939	
SCCmec_type_IV(2B)|SCCmec_type_IVc(2B)|gb|AB096217.1	34675	17549	151.00	3.6e-25	9.37	58.30	0.56	59474	
SCCmec_type_IV(2B&5)|gb|AM292304.1	33454	19547	116.10	3.6e-25	9.04	50.50	0.48	66245	
SCCmec_type_IV(2B)|SCCmec_type_IVc(2B)|gb|AJ810121.1	32385	13810	185.00	3.6e-25	8.75	69.20	0.69	46802	

Selected %ID threshold: *90 %*

Selected minimum length: *60 %*

Selected database: *reference*

Input Files: *A0024SA0006_SCC.fasta*

Results | Gene prediction | SCCmec elements

Support | Scientific problems | Technical problems

Copyright DTU 2011 / All rights reserved
Center for Genomic Epidemiology, DTU, Kemitorvet, Building 204, 2800 Kgs. Lyngby, Denmark
Contact: Vibeke Dybdahl Hammer, Telephone: +45 3588 6420, E-mail: vdha@food.dtu.dk
Funded by: The Danish Council for Strategic Research
Last modified May 22, 2012 11:08:01 GMT

Figure S6. SCCmecFinder report of M5.

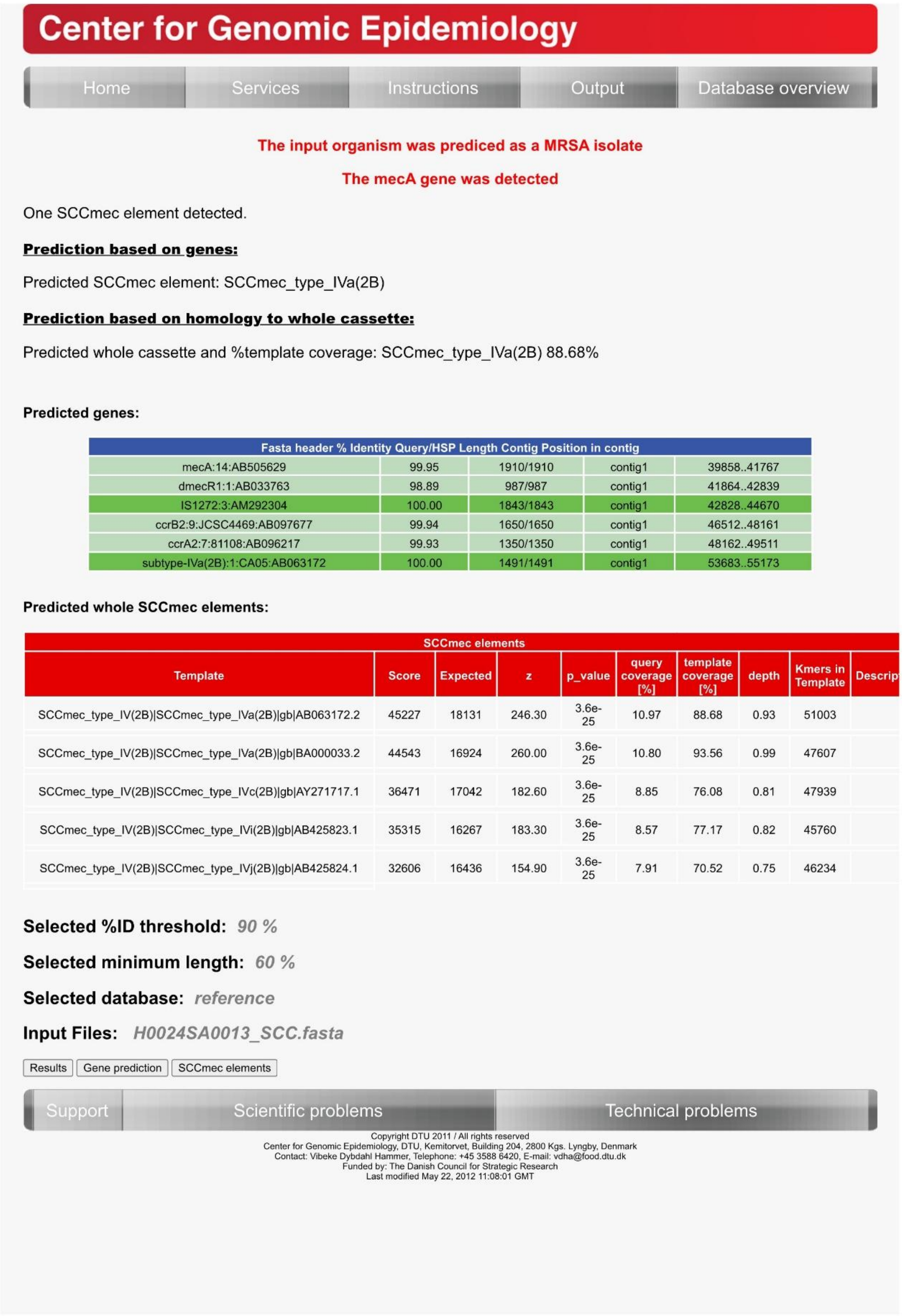

Center for Genomic Epidemiology

Home | Services | Instructions | Output | Database overview

The input organism was prediced as a MRSA isolate

The mecA gene was detected

One SCCmec element detected.

Prediction based on genes:

Predicted SCCmec element: SCCmec_type_IVa(2B)

Prediction based on homology to whole cassette:

Predicted whole cassette and %template coverage: SCCmec_type_IVa(2B) 88.68%

Predicted genes:

Fasta header	% Identity	Query/HSP Length	Contig	Position in contig
mecA:14:AB505629	99.95	1910/1910	contig1	39858..41767
dmecR1:1:AB033763	98.89	987/987	contig1	41864..42839
IS1272:3:AM292304	100.00	1843/1843	contig1	42828..44670
ccrB2:9:JCSC4469:AB097677	99.94	1650/1650	contig1	46512..48161
ccrA2:7:81108:AB096217	99.93	1350/1350	contig1	48162..49511
subtype-IVa(2B):1:CA05:AB063172	100.00	1491/1491	contig1	53683..55173

Predicted whole SCCmec elements:

SCCmec elements									
Template	Score	Expected	z	p_value	query coverage [%]	template coverage [%]	depth	Kmers in Template	Descrip
SCCmec_type_IV(2B)|SCCmec_type_IVa(2B)|gb|AB063172.2	45227	18131	246.30	3.6e-25	10.97	88.68	0.93	51003	
SCCmec_type_IV(2B)|SCCmec_type_IVa(2B)|gb|BA000033.2	44543	16924	260.00	3.6e-25	10.80	93.56	0.99	47607	
SCCmec_type_IV(2B)|SCCmec_type_IVc(2B)|gb|AY271717.1	36471	17042	182.60	3.6e-25	8.85	76.08	0.81	47939	
SCCmec_type_IV(2B)|SCCmec_type_IVi(2B)|gb|AB425823.1	35315	16267	183.30	3.6e-25	8.57	77.17	0.82	45760	
SCCmec_type_IV(2B)|SCCmec_type_IVj(2B)|gb|AB425824.1	32606	16436	154.90	3.6e-25	7.91	70.52	0.75	46234	

Selected %ID threshold: *90 %*

Selected minimum length: *60 %*

Selected database: *reference*

Input Files: *H0024SA0013_SCC.fasta*

Results | Gene prediction | SCCmec elements

Support | Scientific problems | Technical problems

Copyright DTU 2011 / All rights reserved
Center for Genomic Epidemiology, DTU, Kemitorvet, Building 204, 2800 Kgs. Lyngby, Denmark
Contact: Vibeke Dybdahl Hammer, Telephone: +45 3588 6420, E-mail: vdha@food.dtu.dk
Funded by: The Danish Council for Strategic Research
Last modified May 22, 2012 11:08:01 GMT

Figure S7. SCCmecFinder report of M6.

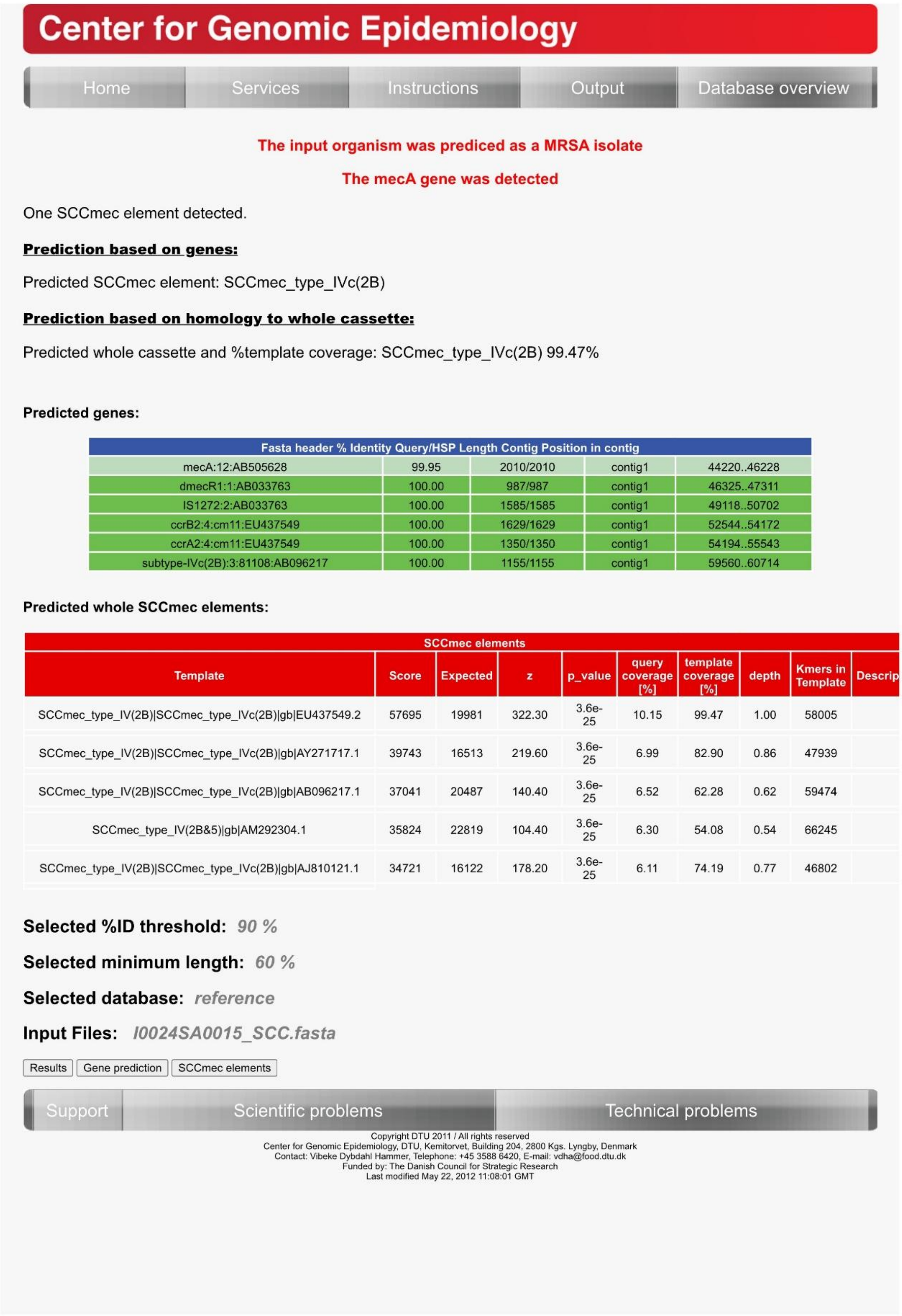

Center for Genomic Epidemiology

Home | Services | Instructions | Output | Database overview

The input organism was prediced as a MRSA isolate

The mecA gene was detected

One SCCmec element detected.

Prediction based on genes:

Predicted SCCmec element: SCCmec_type_IVc(2B)

Prediction based on homology to whole cassette:

Predicted whole cassette and %template coverage: SCCmec_type_IVc(2B) 99.47%

Predicted genes:

Fasta header	% Identity	Query/HSP Length	Contig	Position in contig
mecA:12:AB505628	99.95	2010/2010	contig1	44220..46228
dmecR1:1:AB033763	100.00	987/987	contig1	46325..47311
IS1272:2:AB033763	100.00	1585/1585	contig1	49118..50702
ccrB2:4:cm11:EU437549	100.00	1629/1629	contig1	52544..54172
ccrA2:4:cm11:EU437549	100.00	1350/1350	contig1	54194..55543
subtype-IVc(2B):3:81108:AB096217	100.00	1155/1155	contig1	59560..60714

Predicted whole SCCmec elements:

SCCmec elements									
Template	Score	Expected	z	p_value	query coverage [%]	template coverage [%]	depth	Kmers in Template	Descrip
SCCmec_type_IV(2B)|SCCmec_type_IVc(2B)|gb|EU437549.2	57695	19981	322.30	3.6e-25	10.15	99.47	1.00	58005	
SCCmec_type_IV(2B)|SCCmec_type_IVc(2B)|gb|AY271717.1	39743	16513	219.60	3.6e-25	6.99	82.90	0.86	47939	
SCCmec_type_IV(2B)|SCCmec_type_IVc(2B)|gb|AB096217.1	37041	20487	140.40	3.6e-25	6.52	62.28	0.62	59474	
SCCmec_type_IV(2B&5)|gb|AM292304.1	35824	22819	104.40	3.6e-25	6.30	54.08	0.54	66245	
SCCmec_type_IV(2B)|SCCmec_type_IVc(2B)|gb|AJ810121.1	34721	16122	178.20	3.6e-25	6.11	74.19	0.77	46802	

Selected %ID threshold: *90 %*

Selected minimum length: *60 %*

Selected database: *reference*

Input Files: *I0024SA0015_SCC.fasta*

Results | Gene prediction | SCCmec elements

Support | Scientific problems | Technical problems

Copyright DTU 2011 / All rights reserved
Center for Genomic Epidemiology, DTU, Kemitorvet, Building 204, 2800 Kgs. Lyngby, Denmark
Contact: Vibeke Dybdahl Hammer, Telephone: +45 3588 6420, E-mail: vdha@food.dtu.dk
Funded by: The Danish Council for Strategic Research
Last modified May 22, 2012 11:08:01 GMT

Figure S8. SCCmecFinder report of M7.
